# The mechanism of neurofeedback training for treatment of central neuropathic pain in paraplegia: a pilot study

**DOI:** 10.1186/s12883-015-0445-7

**Published:** 2015-10-13

**Authors:** Muhammad Abul Hassan, Matthew Fraser, Bernard A. Conway, David B. Allan, Aleksandra Vuckovic

**Affiliations:** Rehabilitation Engineering and Assistive technologies, Biomedical Engineering Research Division, University of Glasgow, Glasgow, UK; Department of Biomedical Engineering, NED University of Engineering and Technology, Karachi, Pakistan; Queen Elizabeth National Spinal Injuries Unit, Southern General Hospital, Glasgow, UK; Department of Biomedical Engineering, University of Strathclyde, Strathclyde, UK; Biomedical Engineering Research Division, School of Engineering, University of Glasgow, James Watt building (south), G12 8QQ Glasgow, UK

**Keywords:** Central neuropathic pain, neurofeedback, electroencephalography, paraplegia

## Abstract

**Background:**

Central neuropathic pain has a prevalence of 40 % in patients with spinal cord injury. Electroencephalography (EEG) studies showed that this type of pain has identifiable signatures, that could potentially be targeted by a neuromodulation therapy. The aim of the study was to investigate the putative mechanism of neurofeedback training on central neuropathic pain and its underlying brain signatures in patients with chronic paraplegia.

**Methods:**

Patients’ EEG activity was modulated from the sensory-motor cortex, electrode location C3/Cz/C4/P4 in up to 40 training sessions Results. Six out of seven patients reported immediate reduction of pain during neurofeedback training. Best results were achieved with suppressing Ɵ and higher β (20–30 Hz) power and reinforcing α power at C4. Four patients reported clinically significant long-term reduction of pain (>30 %) which lasted at least a month beyond the therapy. EEG during neurofeedback revealed a wide spread modulation of power in all three frequency bands accompanied with changes in the coherence most notable in the beta band. The standardized low resolution electromagnetic tomography analysis of EEG before and after neurofeedback therapy showed the statistically significant reduction of power in beta frequency band in all tested patients. Areas with reduced power included the Dorsolateral Prefrontal Cortex, the Anterior Cingulate Cortex and the Insular Cortex.

**Conclusions:**

Neurofeedback training produces both immediate and longer term reduction of central neuropathic pain that is accompanied with a measurable short and long term modulation of cortical activity. Controlled trials are required to confirm the efficacy of this neurofeedback protocol on treatment of pain. The study is a registered UKCRN clinical trial Nr 9824.

## Background

Central neuropathic pain (CNP) is caused by an injury to the somato-sensory system with a high prevalence in amputation [1], spinal cord injury [2], multiple sclerosis [3], Parkinson disease [4] and stroke [5]. CNP symptoms do not respond well to medications and the drugs used to treat this type of pain are often associated with significant adverse effects [6]. This has generated interest in nonpharmacological treatments based on neuromodulation and neurostimulation, such as hypnosis, meditation, neurofeedback [7], repetitive Transcranial Magnetic Stimulation (rTMS) and transcranial Direct Current Stimulation (tDCS) [8, 9].

Neuroimagining studies have confirmed that neuromodulation techniques such as hypnosis and meditation, can globally influence pain matrix [7, 10]. Neurostimulation techniques such as rTMS and tDCS typically target primary motor cortex, thereby sending inhibitory signals directly to thalamus and reducing the perceived sensation of pain [11].

Neurofeedback is a type of biofeeback in which patients are provided information about their brain activity in a visual or auditory form. It is believed that neurofeedback facilitates global brain connectivity and leads to neuroplasticity [12].

Neurofeedback has been used for treatment of chronic pain, such as complex regional pain syndrome [13], fibromyalgia [14], migraine [15]. Neurofeedback studies for treatment of CNP are inconclusive with respect to the optimal training protocol [16], most likely due to the small number of treatment sessions. Most neurofeedback protocols for chronic pain target the temporal or central area of the cortex, up-regulating EEG activity in the lower β or α band and down-regulating the activity in the Ɵ and higher β band [13–16]. Based on feedback information patients can be trained to voluntarily decrease brain activity thought to be associated with pain processing.

During neurofeedback for treatment of chronic pain, EEG is typically measured at the training site only, providing no evidences of global modulation of EEG during training. Likewise, there is also the lack of evidence whether prolonged neurofeedback treatment produced long-term changes in cortical activity.

Previous research studies have shown that CNP affects resting state EEG causing increase in theta power and the shift of dominant alpha frequency towards the lower range [17, 18]. Several fMRI studies have shown a correlation between CNP and reorganisation of the sensory and motor cortex [19, 20], where, due to sensory loss caused by the injury, the affected cortical somatotopy undergoes re-mapping or reorganisation [20]. Furthermore, fMRI studies showed that during motor imagery patients with pain activate both brain areas related to control of movement and to pain processing [21].

In a recent study of our group [22] we showed that CNP not only modulates the resting state EEG but also affects the evoked response over the sensory-motor cortex during imagined movement of ‘painful’ as well as non-painful limbs in Ɵ, α and β frequency range. Paraplegic patients with CNP had stronger EEG responses during imagined movement than paraplegic patients with no pain and able-bodied people. All of these studies indicate a close relation between the existence of CNP and ‘over activation’ of the sensory-motor cortex.

We therefore hypothesize that similar to rTMS and tDCS, neurofeedback can target the motor cortex, resulting in normalization of evoked responses and reduction of CNP. We propose neurofeedback training for treatment of CNP in chronic paraplegic patients setting three objectives:

(I) Testing the immediate and longer term effect of neurofeedback training on CNP,
(II) Understanding the putative mechanism through which neurofeedback induces wide-spread changes of EEG activity during neurofeedback training,
(III) Assessing the long term effect of training on all cortical structures involved in processing of pain.

## Methods

### Patients

Seven chronic patients with paraplegia (age 50 ± 4, 6 males, 1 female) having CNP under the level of injury were recruited for the study from a cohort of 10 patients recruited for our previous study [22]. The neurological level of Spinal Cord Injury (SCI) was determined using the American Spinal Injury Association (ASIA) Impairment Classification [23]. Inclusion criteria were: paraplegia at level T1 or lower, at least one year post-injury, a treatment history of CNP for at least 6 months and a report of pain level ≥ 5 on the Visual Numerical Scale (0 = no pain, 10 = worst pain imaginable). The general exclusion criteria were: presence of any chronic (non CNP) or acute pain at the time of the experiment; brain injury or other known neurological condition. Patients receiving pharmacological treatment were instructed not to change medications during the neurofeedback therapy. Information about patients is provided in Table 1.

**Table 1:** Information about patients and outcome results of the neurofeedback therapy

Patient	Level of injury/ASIA	Years with injury/pain	Location of pain	Sensation of pain	Medication
P1	T8 A	7/7	abdomen, legs, buttock, fee	pricking, stabbing	G
P2	T7 A	7/7	shanks, feet	burning	P
P3	T6/T7 D	9/9	legs, feet	Pricking, stabbing	P
P4	T6/T7 B	25/24	Abdomen, legs, buttock, feet	Squeezing, stabbing	P
P5	T8 B	9/9	Buttock, legs, feet	Burning, stabbing	P
P6	T5/6 A	11/11	Left leg and foot	burning	G
P7	T12 B	33/4	Legs, feet	Tingling	p

At the beginning and at the end of the study patients filled out the Brief Pain Inventory [24]. Prior to the study they filled out a 7 Point Guy/Farrar Global Impression of Change [25] to test whether pain intensity was stable over the past week. Ethical approval was obtained from the West of Scotland National Health Service for the Greater Glasgow and Clyde Ethical Committee. Informed consent for participation and publication of the study was obtained from the participants. The procedures followed were in accordance with the Helsinki Declaration, Ethical principles for medical research involving human subjects.

### EEG recording

During neurofeedback training patient’s EEG was recorded using 16 channel Usbamp, (Guger technologies, Austria). Sampling frequency was 256 Hz and electrode impedances were below 5 kΩ. The neurofeedback treatment was provided from one electrode at the time but up to 16 electrodes (F3, Fz, F4, T7, C3, Cz, C4, T8, Cp3, CPz, C4, P4, P3, O1, Oz and O2) were recorded simultaneously. The ground electrode was placed on the mastoid on the training side and the reference electrode was placed on the opposite side.

Multichannel EEG recording for off-line analysis was performed before the first training day and after the last training day, from 61 channels (Synamp^2^, Neuroscan, USA) with electrodes placed according to standard 10/10 locations [26] using an ear-linked reference and AFz as ground. Sampling frequency was 250 Hz and impedance was kept below 5 kΩ.

### Real-time data acquisition and analysis

Real time data acquisition and processing was performed with g.RTanalyzer (Guger technologies, Austria) in Simulink, Matlab (Mathworks, USA). A graphical user interface was developed in LabView (National Instruments, USA).

To calculate EEG power in selected frequency bands, EEG of each channel was bandpass filtered (5^th^ order IIR Butterworth) in the selected bands and was then squared and smoothed/averaged over a half second sliding window, updated after each sample, to obtain the bandpower features.

### Neurofeedback protocol

A daily neurofeedback session started and finished with measuring the baseline EEG activity for 2 min in relaxed open eyes and in closed eyes state. One daily neurofeedback training session consisted of 2 sub-sessions with an audio feedback followed by 6–7 sub-sessions with a visual feedback. Each sub-session lasted 5 min.

Training started with an audio neurofeedback provided from the occipital region (Oz) with patients having their eyes closed, for relaxation purposes. Training parameters were calculated in the lower α band (7–10 Hz) at Oz to account for lower peak frequency in patients with CNP [17, 18, 22]. A relative power was calculated with respect to 2–30 Hz band. Patients were trained to increase the α band power with a threshold set at 110 % of the baseline value. Patients listened to relaxing music, which had two levels: quieter when the α power was above a set threshold and louder when the α power was under the threshold.

Following the audio training patients were provided with a visual neurofeedback as a therapy to reduce pain. EEG power was calculated in θ (4–8 Hz), α (9–12 Hz), lower β (12–15 Hz) and higher β (20–30) Hz bands. The higher β will be referred as β further in the text and 12–15 Hz will be called the Sensory Motor Rhythm (SMR). Relative power was calculated with respect to 2–30 Hz band.

Contingencies were set such that increases in the α or SMR and decreases in the Ɵ and β were reinforced. Increased Ɵ band power was found positively related to the presence of CNP [17, 18, 22, 27] and was confirmed in this particular group of patients [22]. The β band power was suppressed because these oscillations are thought to be positively associated with pain [17]. We trained patients to increase either the SMR or α power because they showed promising results in some previous neurofeedback studies on chronic pain including CNP [16, 28]. The group of patients included in the study had the dominant α frequency on average 1 Hz lower than patients with no CNP [22]. Therefore we trained patients to reinforce the energy of EEG signal in a slightly higher α band (9–12 Hz) which does not include lowest α band frequencies at 8Hz. We set the training ‘threshold’ to 110 % of the average power in the α/SMR band and to 90 % of the average power in the Ɵ and β band. Training was provided from the electrodes located over the primary motor and sensory cortex C4/C3/Cz/ P4, one electrode at the time, order as shown in Fig 2. These electrodes were also a preferred stimulation site for rTMS and tDCS [8, 29]. In addition, this group of patients had an ‘overactive’ sensory-motor cortex during a motor imagery task [22] so neurofeedback provided from that area could potentially down-regulate the excessive activity.

**Fig. 1:**
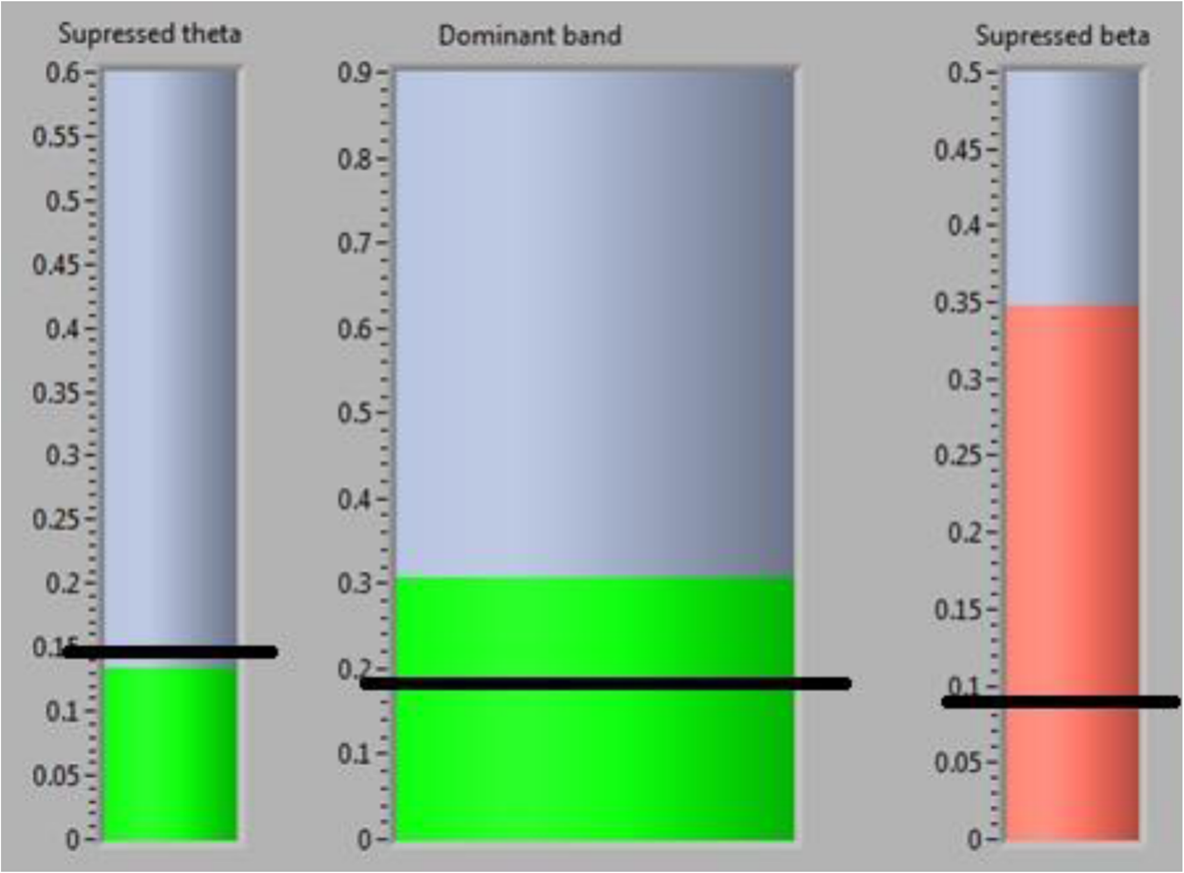
A graphical user interface used for neurofeedback training. Horizontal black lines show threshold values. Central bar shows power of the dominant frequency band, which had green colour when it was reinforced, I,.e. when the power was above the threshold. Side bars present theta (left) and beta (right) frequency bands that were supressed. They had green colour when the power was under the threshold, otherwise were red

During training patients sat in front of a computer screen that showed three bars, the size of which corresponded to the relative EEG power in three chosen frequency bands (Fig. 1). The bars had green colors when the power of a representative frequency band was in the reinforced range and had red color otherwise. The bar in the middle presented a frequency band that had to be rewarded (increased) and turned green when the power was 110 % above the baseline value. Two sidebars presented two frequency bands that had to be inhibited (decreased) and turned green when the power was under 90 % of the baseline value. Patients were instructed to relax and to ‘apply whichever mental strategy they prefer to make the bars green’.

We tested four protocols: *Protocol 1* rewarded SMR and inhibited Ɵ and β at Cz,. *Protocol 2* rewarded α and inhibited Ɵ and β at P4; *Protocol 3* rewarded α and inhibited Ɵ and β at C3. *Protocol 4* rewarded α and inhibited Ɵ and β at C4.

To test for the placebo effect a) patients were shown data from a pre-recorded neurofeedback session, b) they were provided with a visual neurofeedback training to reinforce the α activity at Oz with eyes open; Oz is located at the occipital area, normally not associated with pain.

### Assessment sessions

Resting state EEG was recorded with 61 channels under the open eyes and closed eyes condition in a quiet room. The resting state EEG was recorded for 2 min for each condition repeated 3 times, alternating between the conditions. Assessment sessions were performed twice, up to a week before the first neurofeedback session and up to a week after the last neurofeedback sessions but never on a day of neurofeedback training. Post neurofeedback assessment was performed for patients who had 20 or more neurofeedback sessions.

### EEG off-line analysis

EEG recorded during neurofeedback training was visually inspected and sections containing blinking, muscle activity or amplitude over 100 μV were removed, leaving a minimum of 3 min recording. A power spectral density (PSD) was calculated using Hamming windows over 4 s long recording overlapped for 2 s. Logarithmic PSD was calculated as 10∙log_10_PSD for normalization purposes. For each frequency, the unpaired t-test was performed to compare between two conditions and Holms-Bonferroni correction was applied to reduce the Type I error due to multiple comparisons.

To calculate changes in connectivity during a training session compared to the baseline, coherence was calculated for each channel pair for 4 s long EEG epochs. The average coherence value was calculated for a chosen frequency band. The same statistical analysis was used as for PSD.

Linear regression analysis *Y* = *K*_1_ + *K*_2_ ⋅ *X* was performed to find the best fit curve between the pain intensity and the number of training sessions using the parametric Pearson test.

For 61 channel off-line EEG analysis, data were re-referenced to the average reference. Noise was removed as described above. The current source density was calculated for 4 s long epochs in the Ɵ, α and β band. Localisation of the cortical three-dimensional distribution of the current density of EEG was performed using the Standardised Low Resolution Electromagnetic Tomography sLORETA [30]. The sLORETA method has been shown to have no localization bias [31]. The sLORETA cortical map/image was computed for 6239 voxel partitions of intracerebral volume at 5 mm spatial resolution. Brodmann areas are reported using the Montreal Neurological Institute (MNI) space with correction to the Talairach Space.

For a group level comparison, data of each patient were normalised prior to averaging. A paired t-test was applied to find a statistically significant difference between EEG before and after the therapy. A 5000 voxel randomization of statistical non-parametric mapping [32] implemented in sLORETA package was used to calculate a corrected critical thresholds and p-values.

## Results

Five out of seven patients with pain (P1-5) completed the study. Two patients withdrew after 3 sessions, one because of problems with transportation, although experiencing reduction of pain (P 6) and the other because of the lack of response (P7). Four patients received 40 treatment sessions and the fifth patient, who stayed at the hospital for the purpose of the study, received 20 sessions. Patients who travelled from their homes received 1–3 sessions per week while the patient who stayed at the hospital received five sessions per week.

**Fig. 2:**
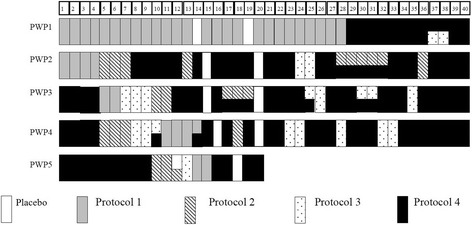
The number of sessions and sequence of training for each protocol

None of patients reported reduction of pain during training with Protocol 1. Patients P2 and P4 reported the moderate reduction of pain as a result of training with Protocol 2. Both Protocols 3 and 4 resulted in a substantial reduction of pain, going down to 0–2 on the visual numerical scale during training. However, patients P3-5 reported strong spasm during training with Protocol 3, which manifested as uncontrollable movements of their paralyzed legs while sitting in front of the computer screen practicing neurofeedback. Patients were trained with Protocol 4 on most of training days as it provided best relief from pain with minimum side effects. Fig. 2 shows the number of sessions and sequence of training for each protocol. The order of protocol was not identical for each patient and depended on their response (no reduction of pain, spasm, etc.).One some days patients were trained with two protocols (3 successive, 5 min long sub-sessions with each protocol). For each training protocol a patient was initially trained for at least two days in a raw, to allow some time to learn neurofeedback strategy for each protocol. Though Protocol 4 seemed to achieve best reduction of pain, other protocols were occasionally re-tested to test whether patients responded in a consistent way. Note that there was no established successful protocol for treatment of CNP, so having an initial hypothesis that the sensory-motor area is overactive in CNP [21, 22] we tested different location over that area of the cortex, keeping similar training parameters. Initial testing of different location is a standard practice in creating a novel neurofeedback protocol [13, 16].

The effect of placebo training was tested on two days, between 10^th^ and 20^th^ training day. The effect of mental rehearsal of neurofeedback was tested within the last 5 training days.

### The effect of neurofeedback training on the intensity of pain

Six out of seven patients reported the immediate reduction of pain already during 2^nd^ or 3^rd^ daily treatment. Initially however that reduction was short-term, during neurofeedback. All five patients who received 20 or more treatment sessions achieved a statistically significant reduction of pain, being clinically significant (>30 % of the individual initial pain intensity as defined by a Visual numerical scale) in four patients (Table 2). The long-term reduction of pain (which lasted beyond the neurofeedback treatment) in all patients was gradual and lasted for several weeks after termination of the therapy. Patients were contacted about one month after the end of the treatment, they still had reduced intensity of pain but it increased 1–2 grades of Visual numerical scale as compared to last neurofeedback session. A linear regression analysis showed a significant negative correlation between the intensity of pain and the number of training sessions (P1: r = 0.74, p = 0.023, P2: r = 0.66, p = 2.5 · 10^−5^; P3: r = 0.61, p = 0.001; P4: r = 0.83, p = 6.85 · 10^−8^; P5: r = 0.64, p = 0.005).

**Table 2:** Information about the outcome of the neurofeedback therapy

Patient	Pain before therapy (VNS)	Pain following therapy (VNS)	Number of sessions
P1	6	5	40
P2	7	5	40
P3	6	2	40
P4	9	6	40
P5	9	6	20
P6	8	6	3
P7	6	6	3

Two patients who before treatment suffered from spasticity/clonus (P3) and spasm/tightness (P4) reported the reduction of this symptoms on the days of training. All patients who experienced the reduction of pain, including patients with the complete loss of sensation, reported the pleasant sensation of warmth in their legs which preceded pain relief and lasted for several hours.

**Fig. 3:**
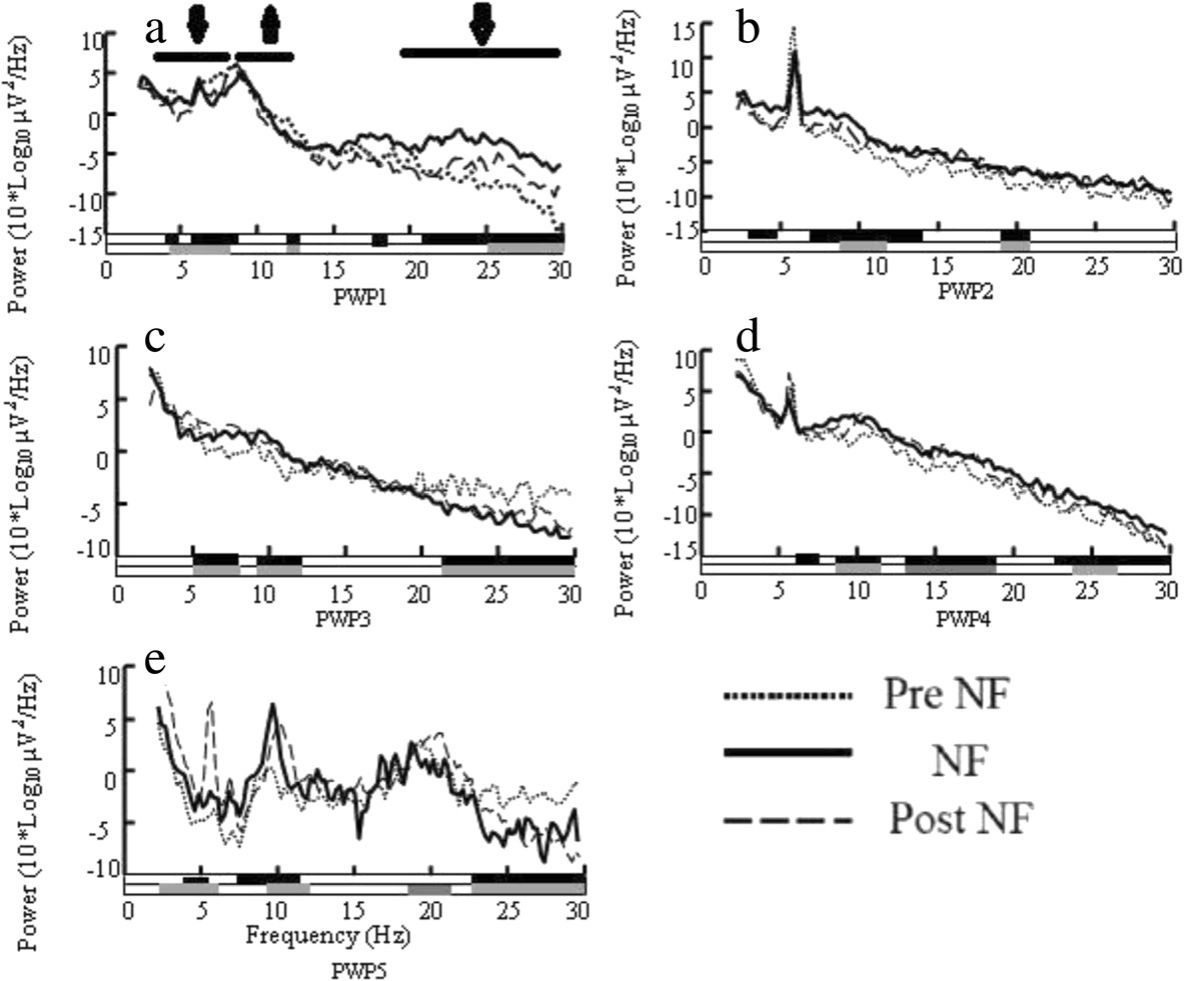
Neurofeedback training with Protocol 4 in P1-5, EEG power as a function of frequency recorded at C4. EEG power at the baseline (‘Pre NF’), during neurofeedback training (‘NF’) and 2–3 minutes following training (‘Post NF’). Bars above graphs show three frequency bands in which training was provided. Arrows up marks a band that was reinforced while arrows down mark frequency bands which were suppressed. Black bars under graphs show statistically significant difference for ‘NF’-‘Pre NF’, while grey bars show statistically significant difference for ‘Post NF’-‘Pre NF’

Patients P2-5 were able to self-regulate their brain activity between treatment sessions (documented by EEG recordings) which helped reducing their pain. An example for P3 is shown in Fig 3b. However they gradually lost that ability a month after the last session due to the lack of a proper visual association.

### EEG modulation during training at the training site

Figure 3a-e show the logarithmic PSD in patients P1-5 before, during and after training on one representative training sub-session lasting 5 min. Patients were able to selectively reinforce or suppress power in different frequency bands, though only P5 modulated power of all three frequency bands simultaneously in a desired direction. It can be noticed that the effect of training on EEG power still remains in the first few minutes following the training (‘Post NF’). Pannel labeled with PWP1 at the bottom corresponds to PSD in patient 1. Consequtive pannels labeled with PWP2,3,4 and 5 corresponds to patients 2,3,4 and 5 respectivly.

**Fig. 4:**
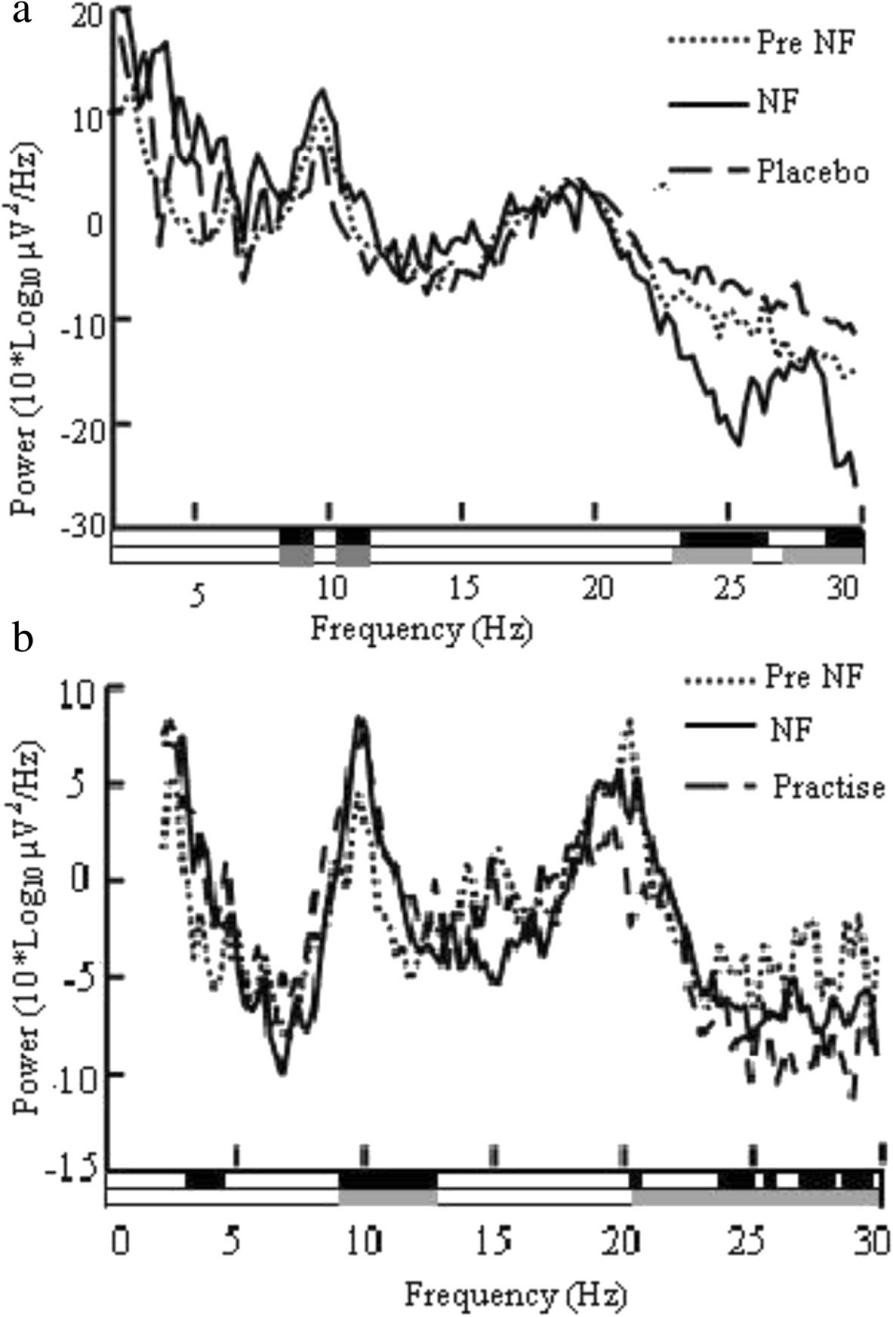
The effect of (**a**) placebo training and (**b**) of mental practice with no visual feedback. Graphs present EEG power as a function of frequency recorded at C4 for both conditions. Fig. 4a shows EEG power in patient P5 during neurofeedback training (‘NF’), placebo training (‘Placebo’) and pre-training baseline (‘Pre NG’), Fig. 4b shows EEG power in patient P5 during neurofeedback (‘NF’), mental practice without a feedback (‘Practice’) and pre-training baseline (‘Pre NF’). Black bars under graphs a and b show statistically significant differences for ‘NF’-‘Pre NF’, while grey bars show statistically significant difference for ‘Pre NF’-‘Placebo’ in (**a**) and for ‘Pre NF’-‘Practice’ in (**b**)

Figure 4a shows PSD before training (‘Pre NF’), during real training (‘NF’) and during placebo training (‘Placebo’) with a pre-recorded session in P5. PSD during placebo training was not significantly different from PSD before training and no reduction of pain was reported. During placebo training with a feedback provided from the electrode location Oz patents unsuccessfully tried to reinforce the α band power (as the occipital alpha normally decreases during visual attention) and did not report any reduction of pain. It should be however mentioned that during a neurofeedback training with closed eyes patients successfully increased their occipital α but none of the patients reported the reduction of pain.

Figure 4b shows PSD in P5 before training (‘Pre NF’), during training with a visual feedback (‘NF’) and during mental neurofeedback practice (‘Practice’) without the visual feedback. The patient regulated EEG power in the same direction and in the same frequency band in the α and β range during the training with feedback and during the mental practice. Four patients P2-5 were able to modulate their PSD during mental neurofeedback practice.

### Global EEG modulation during neurofeedback training

**Fig. 5:**
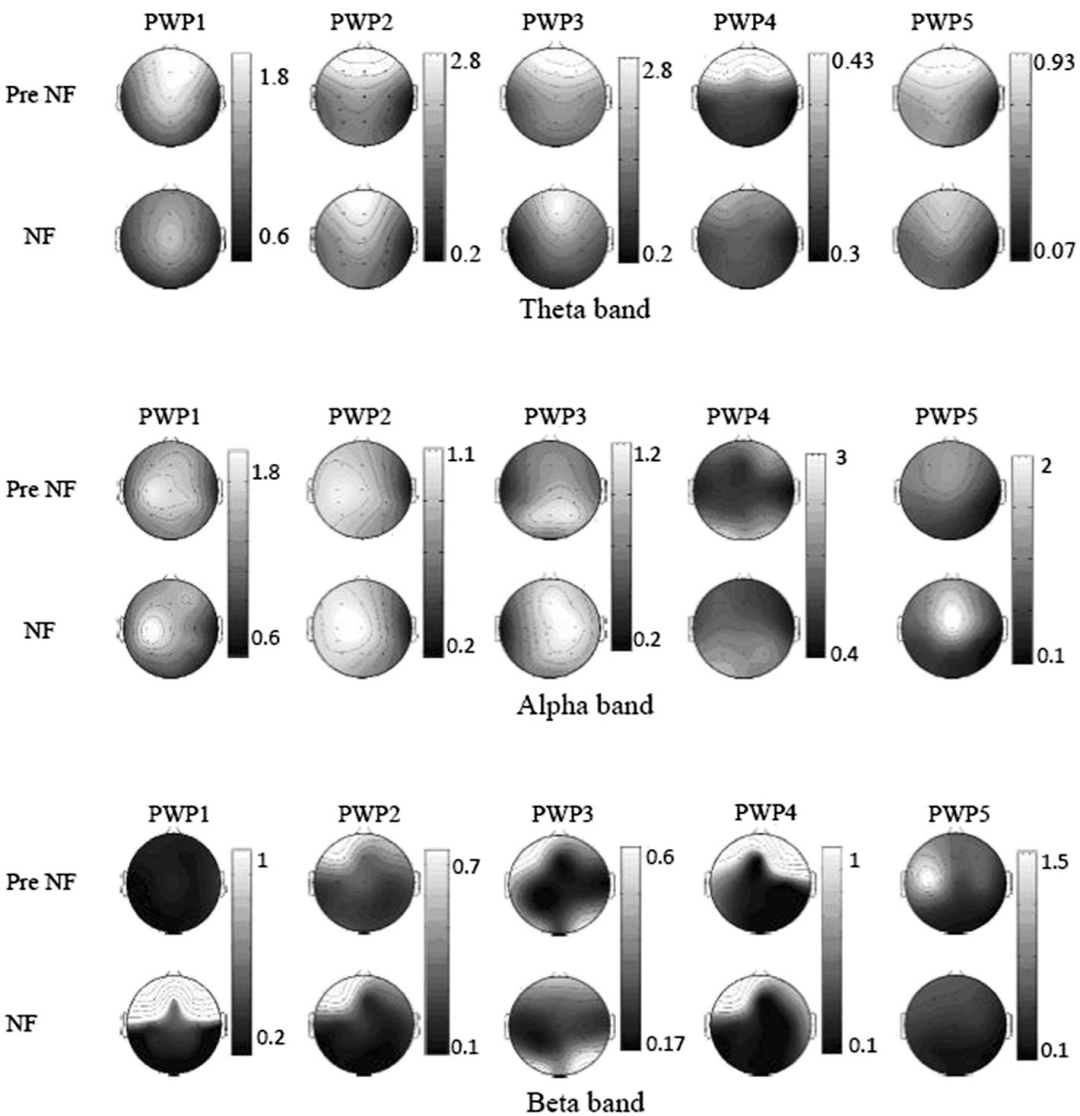
The influence of neurofeedback training with protocol 4 at C4 on wide spread EEG power in different frequency bands. Scalp maps during training (‘NF’) and before training (‘PreNF’) in Ɵ, α and β frequency bands. Location of C4 is shown with black dots in P1 scalp maps. P1-5: patients 1–5. Units on side bar graphs μV^2^

Figure 5 shows scalp maps of PSD over three frequency bands before and during training provided at C4 with protocol 4. Frontal Ɵ was suppressed during training in all five patients. This Ɵ band suppression is likely to be a consequence of voluntary modulation, because increased concentration due to nonspecific engagement in a mental task should result in increased frontal Ɵ [33].

In the α range, training resulted in the increase of power over the central cortex, on both contra and ipsilateral site in P1,2,4 5 while in P3 training resulted in the shift of a maximum α power from the occipital to the central region. In general, the α rhythm characterizes the idle state, and simply focusing attention on object on a computer screen should therefore result in decrease of α power. A wide-spread increase of α power during neurofeedback training could therefore be attributed to the voluntary modulation of brain activity rather than to the general increase of attention. A wide spread increase in the α power can be partially explained by the volume conduction effect, spreading the α activity from the training site at C4. However in P1 and P2 the α power increased more on the contra than on the ipsilateral site of training and in P3 the maximum of α activity shifted from the occipital to the central area.

In four patients (P2-P5) the suppression of the frontal β (20–30 Hz) can be noticed during training, being stronger than suppression at the training site.

**Fig. 6:**
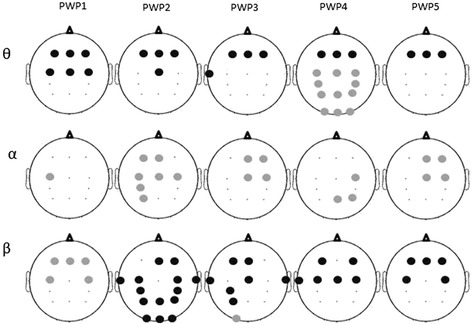
Location of electrodes with significant difference in power (‘NF’—‘PreNF’) shown in Fig 5. Dark circles indicate power increase while grey circles indicate power decrease

Figure 6 shows the location of electrodes with statistically significant differences in PSD between the neurofeedback and the pre-training baseline (PSD neurofeedback- PSD baseline). All patients had a significantly decreased Ɵ band power in the frontal region that in P4 was accompanied with a power increase in the rest of the cortex. All patients had at least one location in the central area of the cortex where the α power significantly increased during training. Four patients (P2-P5) had a significant decrease of β band power in the central and frontal areas. In P2 the decrease of β band power was global and in P1 there was a significant increase of the central and frontal β band power.

**Fig. 7:**
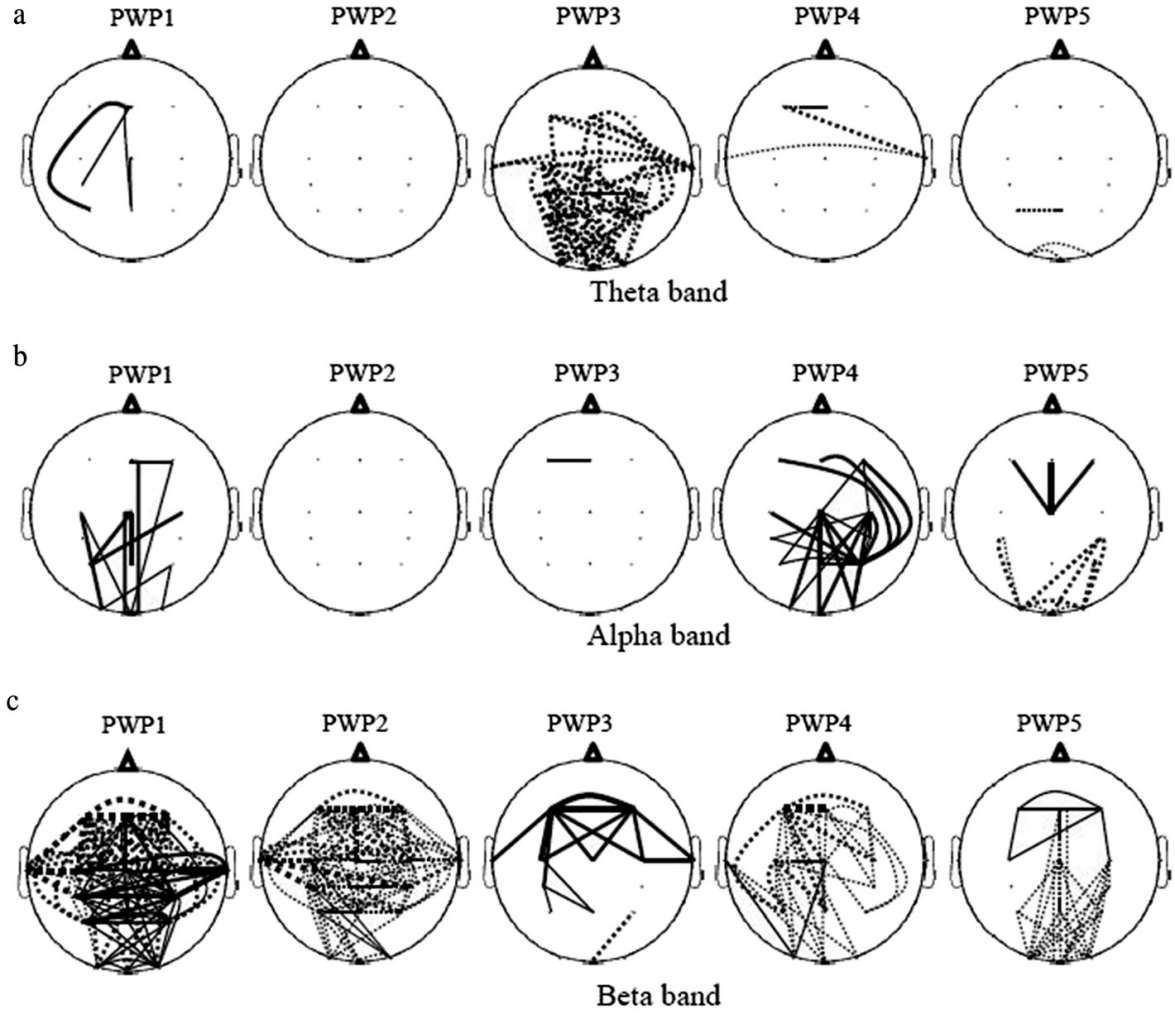
Scalp maps showing statistically significant changes in EEG coherence between 16 scalp sites during training compared to baseline before training (neurofeedback training-baseline) for each patient in three frequency bands (**a**) Theta band, (**b**) Alpha band (**c**) Beta band (significance level p = 0.05, corrected for multiple comparison). Training provided with protocol 4 at C4, marked with a black dot in the upper left figure. The same EEG data used to create EEG power scalp maps in Fig. 5. Solid lines show increase in coherence and dotted lines show decrease in coherence during neurofeedback as compared to the baseline. The thickness of line shows strength of change in coherence (thin line: 0 to 0.1, medium line: 0.1 to 0.2, thick line: 0.2 to onwards)

Figure 7 shows statistically significant difference in coherence (coherence baseline- coherence training) between different cortical regions, in the Ɵ, α and β band (p = 0.05). In the Ɵ band a large decrease in coherence between the occipital and central, occipital and frontal, and central and frontal regions can be noticed only in P 3. In the α band, coherence increased between the occipital and central and the occipital and frontal region in P1 and P4 while in P 5 coherence decreased between the occipital and central region and increased between the central and frontal region. Largest changes in coherence in all five patients were noticed in the β band. In all five patients the coherence decreased between the occipital and central region; in P1,2,4,5 it also decreased between the occipital and frontal areas. In three patients P1,3,5 the interhemispheric coherence increased within the central region.

### Long-term effect of neurofeedback training

**Fig. 8:**
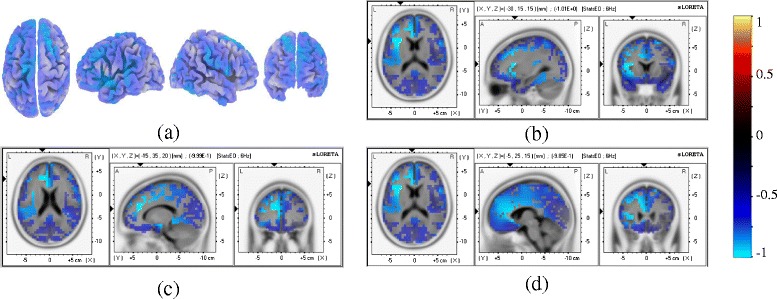
Changes in activity in eyes open state in the β band before the first and after the last day of neurofeedback training (After-Before) averaged over five patients P1-5. (**a**) Surface maps (top, left, right and frontal), (**b**) BA 13 [MNI coordinate: −30 15 15, t = −1.01], (**c**) BA32 [MNI coordinate: −15 35 20, t = −0.99], (**d**) BA 24 [MNI coordinate: −5 25 15, t = −0.99]. Blue colour correspond to reduced activity

A multichannel EEG (61 channles) in a relaxed state was analyzed in the Ɵ, α and β frequency ranges. Statistically significant changes on a group level were noticed in the β band only where reduced activity was noticed in several pain related areas (Table 3). Strongest changes (expressed as the percentage of the total number of voxels) were noticed in the dorso-lateral prefrontal cortex, anterior cingulate cortex and the insular cortex. Fig. 7 shows the reduction of β activity over the surface areas of the cortex (Fig. 8a) and several deeper cortical structures (Fig. 8b-d).

**Table 3:** Percentage of voxels in pain related brain areas showing the average reduced activation

Cortical Areas	BAs	Voxels (%)	Maximum activation	MNI coordinates with maximum value		
S1	1,2,3	NS	/	/	/	/
S2	40,43	NS	/	/	/	/
M1	4	NS	/	/	/	/
PMC	6	2	−0.97	−15	25	40
SMC	8	4	−0.96	−20	30	45
DLPC	9,46	30	−0.99	−15	40	25
APFC	10,11	4	−0.96	−20	45	30
PPC	5,7	/	/	/	/	/
ACC	24,32	20	−0.99	−15	35	20
PCC	23,31	NS	/	/	/	/
IC	13	24	−1.01	−30	15	15

On the individual level, P1 had a significantly reduced activation in the Ɵ band at the primary sensory and motor cortices and the posterior parietal cortex and P2, P4 and P5 had significantly increased α activity over the primary sensory and motor cortices and over all cortical structures reported in table 3. Patient P3, on the contrary, had a significant decrease in the α activity.

## Discussion

This paper presents the effect of neurofeedback training on reduction of CNP and on related neurological measures. Using a visual feedback, patients learned how to modulate their brain activity in a desired directions which resulted in reduction of pain.

Six out of seven tested patients achieved short-term immediate reduction of pain during neurofeedback training. To achieve longer lasting reduction of pain repeated neurofeedback sessions were required. Four out of five patients who received a long term training achieved a clinically relevant reduction of pain (>30 %) lasting at least a month following the treatment. A negative correlation between the intensity of pain and the number of training sessions indicate that the long-term reduction of pain was gradual and required long training. All patients experienced the reduction of pain while receiving a neurofeedback training from electrodes located above the primary motor cortex (C3/C4), which was also a preferred stimulation site for rTMS and tDCS [29, 34]. In Jensen’s et al.,[16] neurofeedback study for treatment of CNP, 10–15 Hz band was reinforced at C3 and C4 while α band was reinforced at T7/T8 with a moderate (not clinically significant) reduction of pain. Better patients’ response in our study might be due to different combination of frequency bands and electrode location (primary motor cortex of legs and arms) or due to the larger number of training sessions, as a prolonged effect of neurofeedback gradually increased over the 40 sessions.

Volunteers from our study that achieved the clinically significant reduction of pain reinforced the α power and to some extent suppressed the β power during training. Although the Ɵ band power is believed to be the signature of CNP [17], the only patient who successfully suppressed the Ɵ rhythm experienced least reduction of pain. This might be related to the fact that patients received medications known to increase the Ɵ band power [35].

Although neurofeedback was dominantly practiced from the right side of the central cortex at C4, patients reported reduction of pain in their legs, confirming observations from rTMS studies that the exact somatotopical location of stimuli is less relevant [36].

Because patients were occasionally trained with different protocols, one cannot claim that training specifically at C4 produced long-term changes. However all protocols involved sensory-motor area and were based on decreasing theta and higher beta band and increasing alpha or lower beta band. In addition, training at one electrode caused wide spread changes so one can assume that all training protocols globally affected sensory-motor cortex, though they caused to some extend different physical responses. In this study, a multichannel EEG was recorded only during training from C4 (Protocol 4). In the future, it would be interesting to compare global effect of training from the electrode locations included in the training protocols (P1-P3).

It is believed that chronic pain disrupts ‘a default mode network’ [37]. Neurofeedback training was accompanied by changes in coherence between occipital and central and occipital and frontal cortex, most notably in the β band. Similar changes in connectivity during hypnosis were attributed to the disruption of pain matrix, possibly establishing a normalized default mode network [7] The higher beta band (20–30 Hz) was the only frequency band in which long-term changes were noticed in all participants. Largest changes occurred in the β band of the dorsolateral prefrontal cortex, the cingulate cortex and the insular cortex. The former is related to the cognitive aspect of pain while two later are parts of the limbic system and deal with the emotional aspect of pain [38]. Previous studies showed that chronic pain shifts brain representation from the nociceptive to the emotional circuits [39], therefore reduction of chronic pain might be first manifested in cortical structures regulating the emotional aspect of pain.

This study tested neurofeedback protocols from the primary motor cortex which is not the part of the standard pain matrix [38]. It is possible that this neurofeedback Protocol 4 is specific to CNP and that it would not be efficient for other types of chronic pain. One of the protocols in this study involved sensory cortex (P4, Protocol 2) but patients reported less reduction of pain than with neurofeedback from C3 and C4. It was difficult to assess how neurofeedback practice with one protocol influenced learning new protocol, though empirically we had impression that it helped, in particular because electrodes were chosen from functionally the same area of the brain. It is hard to measure with EEG the activity of other cortical areas involved in processing of chronic pain but a single daily session fMRI neurofeedback study reported reduced CNP in patients trained to regulate the activity of the anterior cingulate cortex [40].

A perceived sensation of pleasant warmth reported by all patients, including these with a complete loss of sensation, might be an indicator of indirect activation of corresponding thalamic nuclei, being in a close proximity to pain nuclei [41]. Alternatively, it may support the theory of CNP representing a thermoregulatory dysfunction [42].

The ability to ‘self-administer’ therapy i.e. to modulate their brain activity at will without a visual feedback was achieved by 4 out of 5 patients; this ability was previously reported by patients practicing mindfulness and hypnosis [43, 44]. This is a very important observation because CNP in this patients was caused by paralysis and as such can be more or less effectively ameliorated but not cured. Patients ability to self-regulate brain activity on demand would be an important prerequisite for keeping pain under control in long term.

Although some patients reported spasm during training, in two patients this resulted in reduced tightness and clonic activity following neurofeedback. It is believed that sensory-motor cortex is one of the sites from which it is possible to modulate monosynaptic reflexes [45] that might be related to spasm observed in two patients. Previously rTMS stimulation of the primary motor cortex was shown to reduce spasm [46] indicating possible similarities between neuromodulatory mechanism of neurofeedback from sensory-motor cortex and rTMS. To fully understand the potential of the mechanism of neurofeedback treatment, further randomized controlled study is required.

## Conclusions

The results of the study show that prolonged neurofeedback training may have a potential to reduce CNP. Neurofeedback training affects deeper cortical structures involved in processing of chronic pain. We tested neurofeedback treatment on patients who had long-standing CNP. The effect of neurofeedback might be better on patients who suffered from CNP for a shorter period of time, as prolonged pain might cause long lasting changes in brain connectivity 19]. Larger controlled trial would be needed to confirm this results before it could be recommended as a neurofeedback training protocol for CNP.

## Abbreviations

CNP: Central Neuropathic Pain
EEG: Electroencephalography
P: Patient with pain
PSD: Power spectral density
rTMS: repetitive transcranial magnetic stimulation
SCI: Spinal Cord Injury
tDCS: transcranial direct current stimulation
α: alpha band EEG
β: band EEG
θ: band EEG
